# Establishment and application of a rapid assay for GII.4/GII.17 NoV detection based on the combination of CRISPR/Cas13a and isothermal amplification

**DOI:** 10.3389/fmicb.2024.1334387

**Published:** 2024-02-08

**Authors:** Jia-Heng Li, Duona Jing, Yu Wang, Jiayi Xu, Junxuan Yu, Huisha Du, Qing Chen, Shixing Tang, Xu-Fu Zhang, Ying-Chun Dai

**Affiliations:** ^1^Guangdong Provincial Key Laboratory of Tropical Disease Research, Department of Epidemiology, School of Public Health, Southern Medical University, Guangzhou, China; ^2^The Fifth Affiliated Hospital, Southern Medical University, Guangzhou, China; ^3^School of Traditional Chinese Medicine, Southern Medical University, Guangzhou, China

**Keywords:** norovirus, genotype GII.4/GII.17, CRISPR/Cas13a, RAA, detection

## Abstract

**Introduction:**

Norovirus (NoV) is one of the most important agents responsible for viral acute gastroenteritis, among which GII.4 NoV is the predominant strain worldwide, and GII.17 NoV surpassed GII.4 in some epidemic seasons. Rapid and accurate gene recognition is essential for a timely response to NoV outbreaks.

**Methods:**

In the present study, the highly conserved regions of GII.4 and GII.17 NoVs were identified in the junction of open reading frame (ORF) 1 and ORF2 and then amplified by isothermal recombinase-aided amplification (RAA), followed by the cleavage of CRISPR-Cas13a with screened CRISPR RNAs (crRNAs) and RAA primers. The entire detection procedure could be completed within 40 min using a thermostat, and the results could be read out by the naked eye under a portable blue light transilluminator.

**Discussion:**

The assay showed a high sensitivity of 97.96% and a high specificity of 100.0%. It offered a low limit of detection (LOD) of 2.5×10^0^ copies/reaction and a coincidence rate of 96.75% in 71 clinical fecal samples. Overall, rapid and inexpensive detection of GII.4/GII.17 NoVs was established, which makes it possible to be used in areas with limited resources, particularly in low-income countries. Furthermore, it will contribute to assessing transmission risks and implementing control measures for GII.4/GII.17 NoVs, making healthcare more accessible worldwide.

## 1 Introduction

Noroviruses (NoVs) are one of the most common causes of acute viral gastroenteritis (AGE), contributing to approximately one-fifth of all AGE cases worldwide (Pires et al., 2015). These viruses are highly infectious and resistant to inactivation (Gregory et al., 2011). With a global estimate, annual reports showed a huge economic burden of US $4.2 billion in direct health system costs and a societal cost of US$60.3 billion. Furthermore, an annual death toll exceeding 200,000 in developing countries was also estimated (Lopman et al., 2016).

NoVs are members of the family *Caliciviridae*, non-enveloped viruses, possessing a single-stranded RNA genome of 7.5–7.7 kb (Atmar and Estes, 2001). Currently, NoVs are classified into 10 genogroups (GI–GX), which can be further divided into at least 49 genotypes (Chhabra et al., 2019). Genogroups GI, GII, and GIV can infect humans. In the past decades, GII.4 NoVs have been the most predominant strain worldwide, accounting for 70–80% of all reported outbreaks (de Graaf et al., 2016; Dey et al., 2021). Meanwhile, the novel GII.P17-GII.17 NoVs strain became the predominant strain, surpassing the GII.4 strain, in Asia during the 2014–2016 NoV epidemic seasons (de Graaf et al., 2015).

Several molecular diagnostic platforms have been developed for the simultaneous detection of NoVs, such as immunochromatography (ICG) and enzyme immunoassays (EIAs). Real-time quantitative PCR (RT-qPCR) assays have become the gold standard for NoV detection in most clinical laboratories (Vinje, 2015). Excellent specificity and sensitivity make RT-qPCR a preferred assay in most clinical settings; however, it is difficult to popularize in low-income countries and regions due to the high cost of the required equipment. A novel molecular assay has exhibited great prospects by combining isothermal amplification with CRISPR-Cas effector 13a, previously called C2c2 (Gootenberg et al., 2017). Cas 13a is an RNA-guide RNase conferring cleavage activity to degrade any single-stranded RNA (ssRNA) upon a specific sequence (longer than 24 nt) on CRISPR RNAs (crRNAs) (Abudayyeh et al., 2016). Once a Cas 13a-crRNA complex is activated by binding to its target RNA, it can cleave any ssRNA substrate, irrespective of the target RNA or other non-specific RNAs. As a result, the novel assay offers rapid and accurate gene recognition at physiological temperature due to the collateral cleavage activity of Cas13a and isothermal amplification (Gootenberg et al., 2017).

Due to the lack of suitable *in vitro* cell culture systems and small animal models, the development of vaccines specific to NoVs as well as antiviral drugs is quite limited, and treatments are currently constrained to supportive therapy. It makes breaking down the spread of NoV transmission more complicated. Rapid diagnosis of both symptomatic and asymptomatic infections can aid a timely response to NoV outbreaks, which will highly reduce the spread of the virus. In the present study, a one-pot GII.4/GII.17 NoV detection assay was developed based on CRISPR-Cas effector 13a and isothermal recombinase-aided amplification (RAA). It affords rapid, economic, and large-scale on-site detection to prevent and control the spread of NoVs.

## 2 Materials and methods

### 2.1 Clinical sample collection and NoV detection

A total of 71 fecal specimens were included, among which 32 were positive to GII.4, 17 positive to GII.17, and 22 fecal samples positive to GI NoVs, human bocavirus (HBoV), Sapovirus (SaV), rotavirus (RV), Adenovirus (AdV), and Enterovirus 71 (EV71), served as negative controls and for specificity assessment. These fecal samples were obtained from patients with AGE, among which NoV samples were collected from the Fifth Affiliated Hospital of Southern Medical University, while the remaining samples were collected by our group previously.

RNA was extracted from stool specimens using the MiniBEST Viral RNA/DNA Extraction Kit Ver.5.0 (TaKaRa, Japan). The complementary DNA (cDNA) was synthesized using the RevertAid First Strand cDNA Synthesis Kit (Thermo Fisher Scientific, United States). All the fecal samples were first detected using one-step RT-qPCR with GI and GII primers (Sandro Wolf et al., 2007), and then samples positive for GII NoVs were further determined using RT-PCR with primers COG2F and GIISKR for further genotyping (Shigeyuki Kojima et al., 2002). The fecal samples positive for HBoV, SaV, RV, AdV, and EV71 were confirmed by nested RT-PCR as described (Zheng et al., 2016). All the positive PCR products were sequenced and blasted using the NCBI nucleotide blast tool.

### 2.2 Construction and purification of the plasmids of GII.4 and GII.17 NoVs

To optimize our assay and evaluate the sensitivity of the RAA/CRISPR-Cas13a assay, a 523 bp fragment covering the connection region of GII.4 ORF1 and ORF2 was inserted into pJET1.2/blunt using the CloneJET PCR Cloning Kit (Thermo Fisher Scientific, United States), which served as the reference plasmid of GII.4 NoV. The plasmid was verified by sequencing, and its concentration was determined by a NanoDrop 2000 spectrophotometer (Thermo Fisher Scientific, United States). An in-house-made plasmid of full-length GII.17 NoV was also used as the reference plasmid of GII.17 NoV and the full sequence was confirmed by sequencing (Beijing Genomics Institution (BGI), Shenzhen, China).

### 2.3 Design and screening of the primers

In total, 12 primers of GII.4 and 13 of GII.17 specifically for the target region were designed according to the manufacturer’s instructions of the RAA nucleic acid amplification kit (Qitian, Jiangsu, China). All primers were synthesized with lengths ranging from 30 to 38 bp (BGI, Shenzhen, China). Each forward primer incorporated a T7 promoter to facilitate transcription. The expected sizes of the amplified products fell within a range of 134–472 bp. All the primer sequences are listed in Table 1.

**Table 1:** Primer designs used for optimization of the RAA/CRISPR-Cas13a assay for GII.4/GII.17 NoV detection.

Name	Sequence
GII.4-1F	TAATACGACTCACTATAGGGTGAGCACGTGGGAGGGCGATCGCAATCTKGCT
GII.4-2F	TAATACGACTCACTATAGGGTTCTCTGATTTGAGCACGTGGGAGGGCGATCG
GII.4-3F	TAATACGACTCACTATAGGGTTTGAGCACGTGGGAGGGCGATCGCAATCT
GII.4-4F	TAATACGACTCACTATAGGGTTCAGATGGATGAGGTTCTCTGACTTGAGCACG
GII.4-1R	GCTCCATAGTATCTCACCTGGAGCGTTTCT
GII.4-2R	TTCCCCGCGAGGATTACCTGCACTTCAAAGCCACC
GII.4-3R	CGACGCCATCTTCATTCACAAAATTGGGAGC
GII.4-4R	GGACCCATCAGATGGGTTGACGTCATTCGAC
GII.4-5R	GCTCCATAGTATYTCACYTGGAGCGTTTCT
GII.4-6R	CCACCAGGGGCTTGTACAAAATTGTTTCTAATCCAGGG
GII.4-7R	GGGGCTTGTACAAAATTGTTTCTAATCCAGGGGTC
GII.17-1F	TAATACGACTCACTATAGGGGATCTAAGCACATGGGAGGGCGATCGCAATCTG
GII.17-2F	TAATACGACTCACTATAGGGATCTAAGCACATGGGAGGGCGATCGCAATCT
GII.17-3F	TAATACGACTCACTATAGGGGTCATATCTGAACTTAAAGAGGGAGGAATG
GII.17-4F	TAATACGACTCACTATAGGGGCTCATGGCACTACTAGGAGAATCYTCCCTA
GII.17-5F	TAATACGACTCACTATAGGGCCTGTGCAGCTCATGGCACTACTAGGAGAATC
GII.17-6F	TAATACGACTCACTATAGGGTCCCTACATGGACCCTCATTTTACAGCAAGG
GII.17-7F	TAATACGACTCACTATAGGGTTCTCAGATCTAAGCACATGGGAGGGCGATCGC
GII.17-8F	TAATACGACTCACTATAGGGTTCAGATGGATGAGGTTCTCAGATCTAAGC
GII.17-1R	CCCTCTGGTACGAGACCAGCAGCACCATCATTAG
GII.17-2R	TTTGGCCAGTGACGGGTGCGGCTATAGCTG
GII.17-3R	TCTCGTTATTGCCCTCTGGTACGAGACCAGCA
GII.17-4R	TTCTGGGTGACACTGTGAACTCTCCATTTGGTGC
GII.17-5R	GGTGCTTGCACAAAATTTGTTCTAATCCAG

To optimize different pairs of primers, RAA reactions were performed with an RAA nucleic acid amplification kit. In brief, the RAA reaction mixtures included 6.25 μL of rehydration buffer [pre-mixed in advance with dried enzyme pellet (including recombinase, single-stranded DNA binding protein, and strand-displacing DNA polymerase)], 0.5 μL of each primer (10 μM), 2 μL of purified plasmid DNA template (2.4 × 10^4^ copies/μL), and 1.25 μL of magnesium acetate (280 mM). After 20 min incubation at 39°C, the results were evaluated using agarose gel electrophoresis. The primer pairs with high performance were selected and used in subsequent experiments.

### 2.4 Design and generation of the crRNAs for GII.4 and GII.17 NoVs

To optimize the accessibility of the target site of crRNAs, a total of 157 GII.4/GII.17 full-length sequences were downloaded from the National Center for Biotechnology Information (NCBI). Seventy-three sequences were GII.4 NoV from more than 20 countries and regions, including different variants, such as GII.Pe-GII.4 (Sydney 2012), GII.P31-GII.4 (Hong Kong), GII.P4-GII.4. Eighty-four sequences were GII.17 NoV from more than 15 countries and regions, including multiple variants, such as GII.17 (Kawasaki-2014) and GII.P17-GII.17. The sequences were aligned to find the most conserved crRNAs binding regions using MEGA 11, then output conserved regions of the alignment using BioEdit. As a result, six crRNAs targeting the conserved region at the junction of ORF1 and ORF2 were obtained. The corresponding DNA oligonucleotides consisted of the T7 promoter, LwCas13a repeated RNA sequence, and guiding sequence. The whole complementary single-stranded DNA was then synthesized (BGI, Shenzhen, China). To form DNA double strands, single-stranded DNAs were denatured at 94°C for 3 min and annealed from 94 to 25°C with a temperature reduction of 2°C every minute. Subsequently, the annealed products were transcribed using a TranscriptAid T7 High Yield Transcription Kit (Thermo Fisher Scientific, United States), incubating at 37°C for 2 h. Transcribed products were purified by the Monarch RNA Cleanup Kit (New England Biolabs, United States) at room temperature. The concentration of the purified crRNAs was quantified using a NanoDrop 2000 spectrophotometer (Thermo Fisher Scientific, United States) and stored at −80°C after aliquoting. All the crRNA sequences are listed in Table 2.

**Table 2:** Design of crRNA used for the optimization of RAA/CRISPR-Cas13a assay for GII.4/GII.17 NoV detection.

Name	Sequence
GII.4-crRNA1	TAATACGACTCACTATAGGGGATTTAGACTACCCCAAAAACGAAGGGGACTAAAACAGATTGCGATCGCCCTCCCACGTGCTCA
GII.4-crRNA2	TAATACGACTCACTATAGGGGATTTAGACTACCCCAAAAACGAAGGGGACTAAAACCGCCATCTTCATTCACAAAACTGGGAGC
GII.4-crRNA3	TAATACGACTCACTATAGGGGATTTAGACTACCCCAAAAACGAAGGGGACTAAAACGACCCATCAGATGGGTTGACGTCA
GII.17-crRNA1	TAATACGACTCACTATAGGGGATTTAGACTACCCCAAAAACGAAGGGGACTAAAACCTGGGAGCCAGATTGCGATCGCCCTCCC
GII.17-crRNA2	TAATACGACTCACTATAGGGGATTTAGACTACCCCAAAAACGAAGGGGACTAAAACTTCGACGCCATCTTCATTCACAAA
GII.17-crRNA3	TAATACGACTCACTATAGGGGATTTAGACTACCCCAAAAACGAAGGGGACTAAAACCTTCATTCACAAAACTGGGAGCCA

For the screening of crRNAs, all of the corresponding target DNA templates were transcribed to obtain a purified target RNA template as an RNA-targeting CRISPR effector, due to the peculiarity of LwCas13a. The RNA screening system included 2 μL of purified target RNA, 1 μM of LwaCas13a (Editgene, Guangzhou, China), 1 μL of crRNA (85 nM), 2 μL of Editgene buffer, 0.5 μL of RNase inhibitor (New England Biolabs, United States), 5 μM of RNA probe reporter (5’-FAM-UUUUUU-BHQ-1-3’), and 3.8 μL of nuclease-free water, which was incubated at 39°C and measured by a fluorescent detector (Qitian, Jiangsu, China). The fluorescence signals were measured at 10 s intervals. The crRNAs with the best performance were selected and used in the following experiments.

### 2.5 Optimization of recombinase-aided amplification/CRISPR-Cas13a assay in one-pot

The RAA reaction was conducted using the RAA basic kit (Qitian, Jiangsu, China) according to the manufacturer’s instructions with some minor modifications. For optimization, a routine sample addition and a modified one were compared. The routine sample addition was called “RAA-T7, crispr”, a mixture of 6.25 μL of rehydration buffer [pre-mixed in advance with dried enzyme pellet (including recombinase, single-stranded DNA binding protein, and strand-displacing DNA polymerase)], 0.5 μL of each primer (10 μM), 2 μL of target DNA template, 2 μL of T7 RNA polymerase (Thermo Fisher Scientific, United States), and 2 μL of NTPs Mix (Thermo Fisher Scientific, United States) in the tube containing and 1.25 μL of magnesium acetate (280 mM). Subsequently, 5.8 μL of CRISPR reaction mixture [8.5 nM of crRNA, 1 μM of LwaCas13a, 2 μL of Editgene buffer, and 5 μM probe reporter (5’-FAM-UUUUUU-BHQ-1-3′)] were added into the cap of the RAA reaction tube and incubated at 39°C for amplification for 20 min. While the modified sample addition was called ‘RAA, T7-crispr’, which moved the T7 RNA polymerase and NTPs Mix to the cap of the RAA reaction tube.

**Figure 1:**
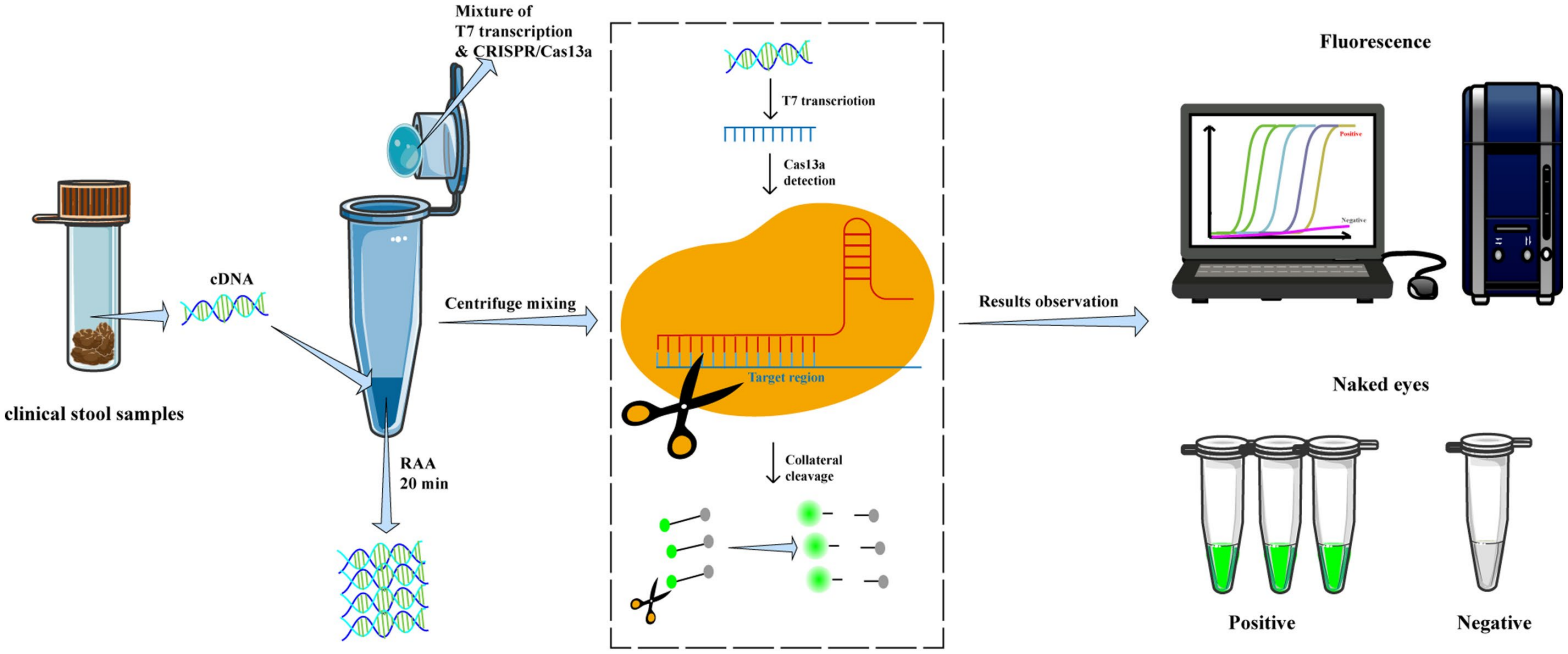
Workflow of RAA/CRISPR-Cas13a for GII.4/GII.17 NoVs detection assay. In brief, viral RNA is extracted from clinical fecal samples and reverse transcripted into cDNA, then amplified via recombinase-based isothermal amplification. The CRISPR/Cas13a reagents are centrifuged and mixed with RAA products to introduce CRISPR/Cas13a cleavage of the amplified products and trans-cleavage of reporter DNA. Finally, the results are measured by a fluorescent detector or read directly by the naked eye under a blue transilluminator.

Afterward, the tube was centrifuged to mix the CRISPR-Cas13a reagents to the bottom of the tube and incubated at 39°C for 30–40 min for detection. The fluorescence signals were measured at 10s intervals using a fluorescent detector (Qitian, Jiangsu, China) in real-time or visually identified under a blue light transilluminator (Figure 1).

## 3 Results

### 3.1 Design and screening of RAA primers

**Figure 2:**
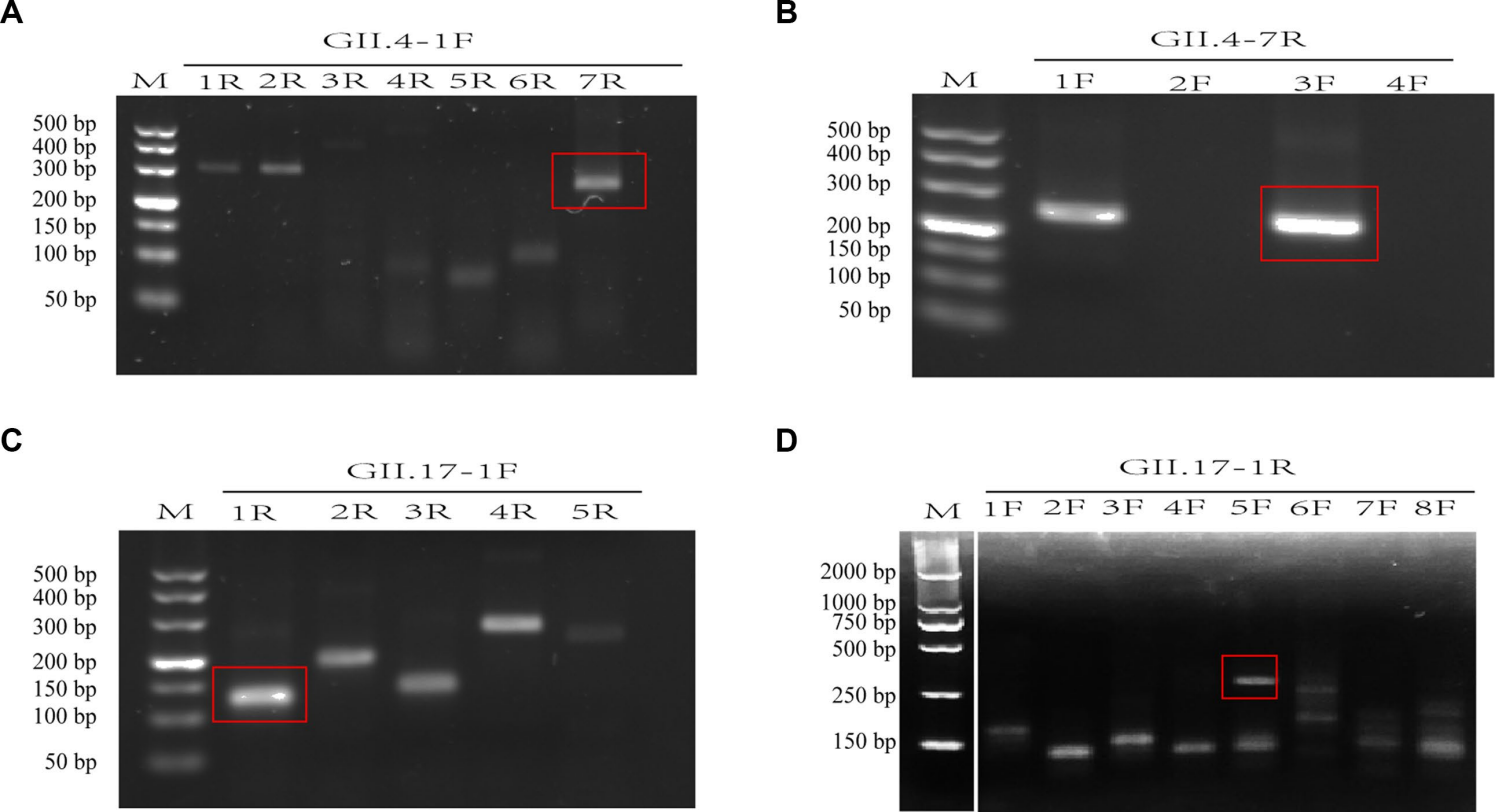
Assessment of primer pairs for recombinase-aided amplification (RAA) using agarose electrophoresis. Brackets indicated optimal bands of expected sizes of RAA products **(A–D)**. M: DNA marker; F: forward primer; R: reverse primer. There is a splice between lanes M and 1F for the cut of positive control band of the RAA kit **(D)**.

A total of 12 primers specific to GII.4 and 13 to GII.17 NoV conserved regions were designed and screened. For GII.4 NoV, three forward and two reverse primers were identified with bright bands of the expected size. Among these, the GII.4-3F/7R primer pair presented the clearest and brightest bands without smearing and therefore was selected for further experiments (Figures 2A,B). For GII.17 NoV, four forward primers and two reverse primers were identified, and primer pair GII.17-5F/1R exhibited the best performance (Figures 2C,D).

### 3.2 Evaluation of CRISPR/Cas13a crRNAs and one-pot assay

**Figure 3:**
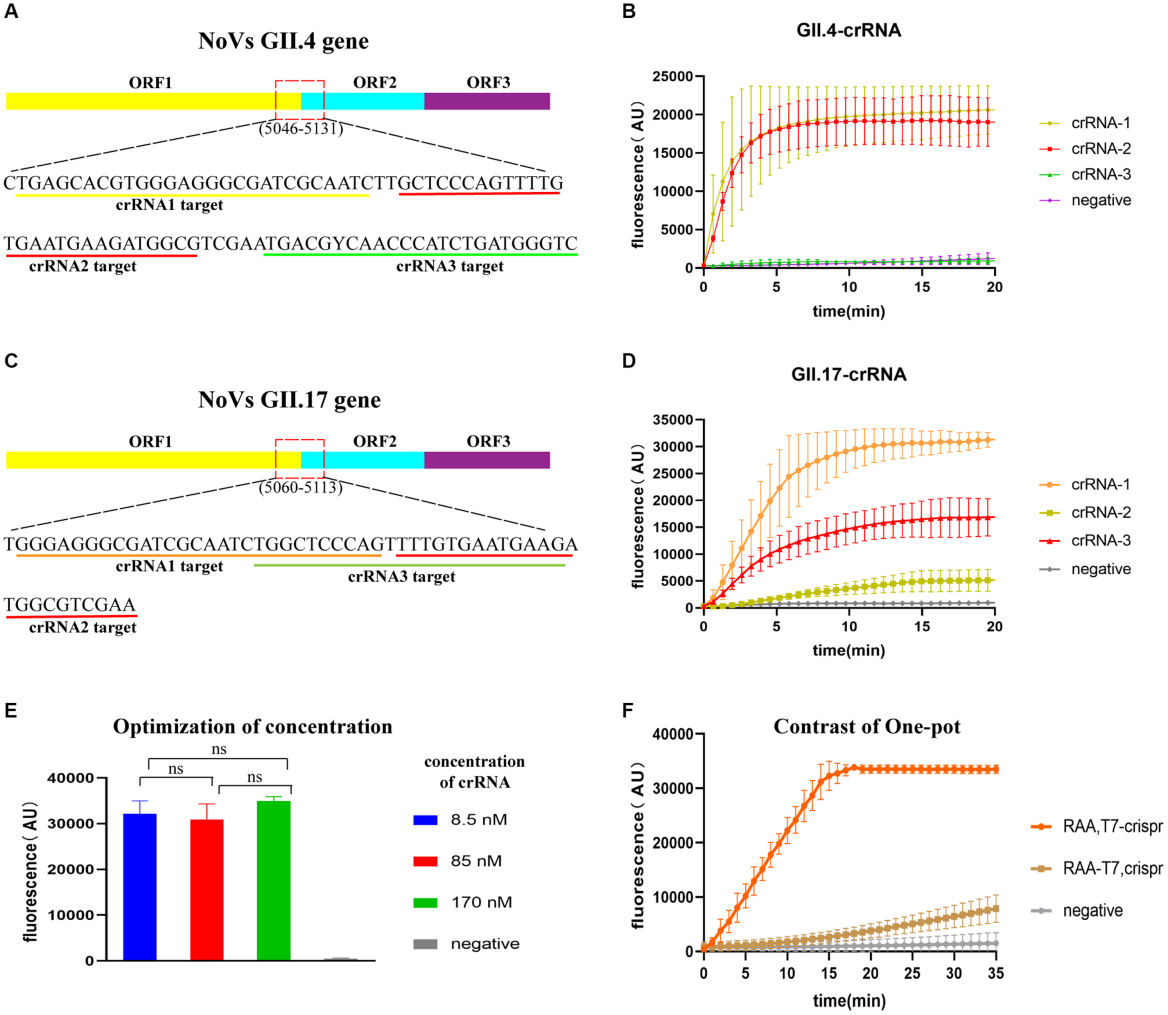
Evaluation of CRISPR/Cas13a crRNAs and one-pot assay. Target regions of crRNA designed for GII.4/GII.17 NoVs **(A,C)** and fluorescence curve for crRNA screening **(B,D)**. Optimization of crRNA concentration, with “ns” representing no statistical difference, *p* > 0.05 **(E)**. Fluorescence curves for two sample addition options (“RAA-T7, Crispr”, and “RAA, T7-Crispr”) **(F)**.

The most conserved regions in the junction of ORF1 and ORF2 (GII.4: 5046–5131; GII.17: 5060–5141) were identified based on 157 GII.4/GII.17 full-length sequences. Three crRNAs were designed for each GII.4 and GII.17 NoV (Figures 3A,C), using an identical concentration for screening. GII.4-crRNA-1 and GII.17-crRNA-1 exhibited superior performance, reaching higher peak efficiency within 10 min (Figures 3B,D). For the chosen crRNA-1, it elucidated a high efficiency of cleavage at a low concentration of 8.5 nM (Figure 3E), which was ultimately selected for the following experiments.

To test the efficiency of one-pot detection, two options of sample addition were tested: “RAA-T7, Crispr” and “RAA, T7-Crispr”. Sample addition in RAA, T7-Crispr reached the peak faster and higher within 20 min (Figure 3F), which was chosen for the following experiments.

### 3.3 Limit of detection (LOD) and specificity of the RAA/Cas13a fluorescence assay

**Figure 4:**
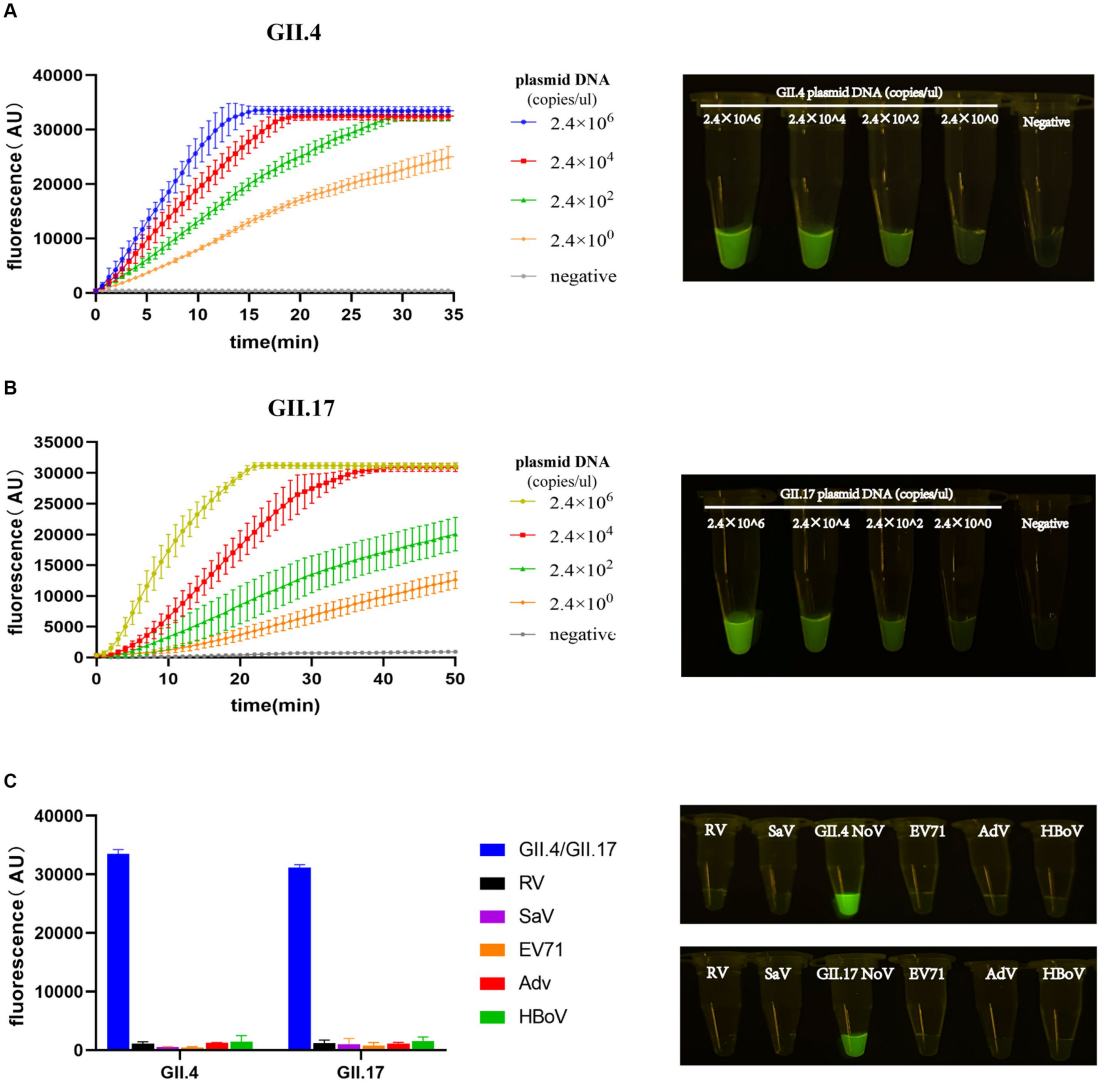
Limit of detection (LOD) and specificity of the RAA/Cas13a fluorescence assay. Fluorescence results of LOD using GII.4/GII.17 plasmid dilution with a 100-fold gradient, along with visual observation under a blue transilluminator at 20 min **(A,B)**. Fluorescence results of specificity, along with the corresponding visual observation **(C)**.

The entire procedure was finalized, including the preparation of experimental materials, the steps of experimental operation, and the RAA primers and crRNAs. To quantify and compare the detection limit performance of our assay with other assays, the LOD value was introduced (Lin et al., 2022). LOD was evaluated by diluting the plasmid DNA template in a 100-fold concentration gradient. The LOD of the RAA/CRISPR-Cas13a assay was confirmed at 2.4 × 10^0^ copies/μL. As the template concentration decreased, the amplitude of fluorescence intensity decreased significantly over time. The procedure of our assay can be performed within 40 min; meanwhile, the lowest template concentration was distinguishable from the negative control group by the naked eye (Figures 4A,B).

To evaluate the specificity of our newly developed assay, five clinical specimens positive for pathogens that have the highest co-infection probability with NoV were selected. Positive results were only detected in the plasmids of GII.4 or GII.17 NoV with a high-intensity fluorescence signal, while all others did not show any fluorescence peaks, demonstrating a specificity of 100% without cross-reactivity to related pathogens (Figure 4C).

### 3.4 Performance of RAA/CRISPR-Cas13a assay in clinical fecal samples

To identify the accuracy of the study results, a total of 71 clinical fecal samples were included. We conducted a comparison of the results obtained from our assay with those obtained by RT-qPCR (RT-PCR) combined with sequencing (Supplementary Table S1). As shown in the results, the sensitivity of the RAA/CRISPR-Cas13a assay was 97.96%, the specificity was 100.00%, and the Kappa value was 96.75%, indicating an excellent coincidence (Table 3).

**Table 3:** Comparison of performance between GII.4/GII.17 RAA/CRISPR-Cas13a assay and sequencing in clinical samples.

RAA/Cas13a detection	Sequencing		Sensitivity (%)	Specificity (%)	Kappa value (%)
	Positive	Negative			
GII.4 positive	31	0	96.9	100.0	97.1
GII.4 negative	1	39			
GII.17 positive	17	54	100.0	100.0	100.0
GII.17 negative	0	0			

## 4 Discussion

NoVs are responsible for more than half of all reported foodborne disease outbreaks, with contaminated food and water being the main culprits (de Graaf et al., 2016). Over the last decade, the repercussions of NoV-induced AGE outbreaks have been widely observed in terms of public health and socioeconomic aspects globally (Mans, 2019). Individuals infected with NoVs have a high level of viral shedding that lasts for a long period of time (Atmar et al., 2008), which makes prevention and control efforts more challenging. Therefore, the development of a convenient and rapid diagnosis is crucial for the prevention and control of NoV outbreaks.

In recent years, as several detection assays of NoV have emerged constantly, RT-qPCR has become the mainstream detection in most clinical settings worldwide. However, RT-qPCR is time-consuming, expensive, and requires expensive equipment, constraining its application in field investigations, especially in developing countries. In contrast, the RAA demonstrates tremendous potential, characterized by its simple operation and fast amplification at a suitable reaction temperature of 37–42°C (Tan et al., 2022). The combination of RAA and CRISPR systems shows great prospects for accuracy, high speed, and convenience. The specific targeting ability of crRNA is crucial in the CRISPR system. Compared to the other CRISPR/Cas12a-based NoV detection methods, Cas13a requires a more relaxed protospacer flanking site (PFS) corresponding to the protospacer adjacent motif (PAM) of Cas12a for its crRNA; thus, avoidance of the G base at the 3′ end is needed (Abudayyeh et al., 2016). Our assay based on CRISPR/Cas13a offers a more attainable condition for the design of crRNAs. RAA combined with CRISPR-Cas13a-based detection can avoid potential specificity reduction caused by RAA primer mismatch with the target sequence, enabling the assay to accurately diagnose with a LOD of 2.4 × 10^0^ copies/μL. We integrated the RAA and T7 transcriptions with CRISPR-Cas13a-based detection into a single tube, which facilitated the simplification of the experimental manipulation procedure and reduced the possible contamination of surrounding nucleic acids. In addition, the entire assay could be completed within 40 min using a thermostat. Furthermore, the results could be read out by the naked eye under a portable blue light transilluminator, making it possible to be conducted in the field and in low-income countries.

For feasible and accurate identification with the target sequence, 157 full-length GII.4/ GII.17 sequences were downloaded from NCBI to carry out multiple sequence alignment. A highly conserved region was identified at the junction of ORF1 and ORF2, for which sets of corresponding crRNAs were designed and screened. Our assay showed a LOD of up to 2.4 × 10^0^ copies/μL, which was significantly higher than the mean viral load in clinical stool samples (3.0×10^5^ copies/μL) (Chan et al., 2006; Anfruns-Estrada et al., 2020). Our assay showed high performance with a coincidence rate of 96.75% and sensitivity of 97.96% and displayed 100% specificity, indicating no cross-reactivity between other analogous AGE pathogens. Accurate diagnosis with high specificity will increase the efficiency of preventing and controlling NoV outbreaks and avoiding unnecessary treatments. Notably, to simplify the experimental manipulation procedures and reduce the contamination of nucleic acid, the assay was optimized for one-pot detection. In our observation, RAA and CRISPR/Cas 13a had to separate in physical space due to the conflict between the nucleic acid amplification and cleavage activity of Cas protein, which was distinct from the description in the other group (Duan et al., 2022). As we know, the cleavage of Cas 13a can reduce the template DNA yield, severely affecting the efficiency of the amplification reaction. In other words, the RAA step needs to be performed at the bottom of the reaction tube, while the CRISPR/Cas 13a step is prepared in the cap of the reaction tube. This can effectively avoid the mutual interference between the amplification process and enzyme cutting, ensuring the accuracy and stability of the reaction. To introduce the T7 transcription system, optimization of sample addition was tested, and our results showed that T7 transcription had a significant impact on RAA amplification but worked in synergy with CRISPR/Cas 13a.

Compared to RT-qPCR, RAA primer design is more complicated, as it requires a length of at least 30 bp resulting in a higher frequency to form the primer dimer, which might affect the amplification efficiency. Meanwhile, the crRNA target site that guides Cas13a requires more than 24 nt, with the optimal length being 28 nt, and the mismatching of a single base may lead to the failure of crRNA targeting, which also brings certain difficulties.

As different concentrations of DNA templates might impact the detection results, no virus loads were determined in the clinical fecal samples, which was a limitation of the present study.

In conclusion, point-of-care pathogen detection of GII.4/GII.17 NoVs was established, which can be completed rapidly, inexpensively, and read with the naked eye, making it possible to be used in areas with limited resources, particularly in low-income countries.

## Data availability statement

The original contributions presented in the study are included in the article/Supplementary material, further inquiries can be directed to the corresponding authors.

## Ethics statement

The studies involving humans were approved by Nanfang Hospital of Southern Medical University. The studies were conducted in accordance with the local legislation and institutional requirements. The participants provided their written informed consent to participate in this study.

## Author contributions

J-HL: Conceptualization, Data curation, Formal analysis, Investigation, Methodology, Software, Visualization, Writing – original draft. DJ: Formal analysis, Writing – review & editing. YW: Formal analysis, Writing – review & editing. JX: Formal analysis, Writing – review & editing. JY: Formal analysis, Writing – review & editing. HD: Formal analysis, Writing – review & editing. QC: Resources, Writing – review & editing. ST: Resources, Writing – review & editing. X-FZ: Funding acquisition, Resources, Writing – review & editing. Y-CD: Conceptualization, Funding acquisition, Project administration, Resources, Supervision, Writing – review & editing.
