# Erratum: Establishment and application of a rapid assay for GII.4/GII.17 NoV detection based on the combination of CRISPR/Cas13a and isothermal amplification

**DOI:** 10.3389/fmicb.2024.1405235

**Published:** 2024-04-02

**Authors:** Frontiers Production Office

**Affiliations:** Frontiers Media SA, Lausanne, Switzerland

**Keywords:** norovirus, genotype GII.4/GII.17, CRISPR/Cas13a, RAA, detection

Due to a production error, the Funding statement was erroneously not included in the final article. The Funding statement is as follows:

