# Supplementary material for: Establishment and application of a rapid assay for GII.4/GII.17 NoV detection based on the combination of CRISPR/Cas13a and isothermal amplification
Source: Front Microbiol. 2024 Feb 8;15:1334387. doi: 10.3389/fmicb.2024.1334387 (PMC10881755; doi:10.3389/fmicb.2024.1334387)
Supplement: Supplementary file 1 [file Table_1.DOCX]

Supplemental table1. The detection result of clinical fecal samples.

| Number | **RT-qPCR** | **Nested-PCR** |
| --- | --- | --- |
| 1 | GI | / |
| 2 | GII | GII.4 |
| 3 | GII | GII.4 |
| 4 | GII | GII.4 |
| 5 | GII | GII.4 |
| 6 | GII | GII.4 |
| 7 | GII | GII.4 |
| 8 | GII | GII.4 |
| 9 | GII | GII.4 |
| 10 | GII | GII.4 |
| 11 | GII | GII.4 |
| 12 | GII | GII.17 |
| 13 | GI | / |
| 14 | GII | GII.4 |
| 15 | GII | GII.4 |
| 16 | GII | GII.4 |
| 17 | GII | GII.4 |
| 18 | GII | GII.4 |
| 19 | GII | GII.4 |
| 20 | GI | / |
| 21 | GI | / |
| 22 | GI | / |
| 23 | GI | / |
| 24 | GII | GII.4 |
| 25 | GI | / |
| 26 | / | RV |
| 27 | GII | GII.4 |
| 28 | GI | / |
| 29 | GII | GII.4 |
| 30 | GII | GII.4 |
| 31 | GII | GII.4 |
| 32 | GII | GII.17 |
| 33 | GII | GII.4 |
| 34 | GII | GII.4 |
| 35 | GII | GII.4 |
| 36 | GII | GII.4 |
| 37 | GII | GII.4 |
| 38 | GII | GII.4 |
| 39 | GII | GII.17 |
| 40 | GII | GII.17 |
| 41 | GII | GII.17 |
| 42 | GI | / |
| 43 | GII | GII.4 |
| 44 | GII | GII.4 |
| 45 | GII | GII.4 |
| 46 | GI | / |
| 47 | GII | GII.4 |
| 48 | GII | GII.17 |
| 49 | GII | GII.4 |
| 50 | / | SaV |
| 51 | GII | GII.17 |
| 52 | GII | GII.17 |
| 53 | GII | GII.17 |
| 54 | GII | GII.17 |
| 55 | GII | GII.17 |
| 56 | / | EV71 |
| 57 | GII | GII.17 |
| 58 | GII | GII.17 |
| 59 | / | SaV |
| 60 | GII | GII.17 |
| 61 | GII | GII.17 |
| 62 | GII | GII.17 |
| 63 | GII | GII.17 |
| 64 | / | HBoV |
| 65 | / | RV |
| 66 | / | RV |
| 67 | / | RV |
| 68 | / | SaV |
| 69 | / | SaV |
| 70 | / | HBoV |
| 71 | / | HBoV |
